# Large magneto-thermopower in MnGe with topological spin texture

**DOI:** 10.1038/s41467-018-02857-1

**Published:** 2018-01-29

**Authors:** Y. Fujishiro, N. Kanazawa, T. Shimojima, A. Nakamura, K. Ishizaka, T. Koretsune, R. Arita, A. Miyake, H. Mitamura, K. Akiba, M. Tokunaga, J. Shiogai, S. Kimura, S. Awaji, A. Tsukazaki, A. Kikkawa, Y. Taguchi, Y. Tokura

**Affiliations:** 1 Department of Applied Physics and Quantum Phase Electronics Center (QPEC), The University of Tokyo, Bunkyo-ku, Tokyo, 113-8656 Japan; 2 RIKEN Center for Emergent Matter Science (CEMS), Wako, Saitama 351-0198 Japan; 3 The Institute for Solid State Physics (ISSP), The University of Tokyo, Kashiwa, Chiba 277-8581 Japan; 4 Institute for Materials Research (IMR), Tohoku University, Aoba-ku, Sendai, 980-8577 Japan

## Abstract

Quantum states characterized by nontrivial topology produce interesting electrodynamics and versatile electronic functionalities. One source for such remarkable phenomena is emergent electromagnetic field, which is the outcome of interplay between topological spin structures with scalar spin chirality and conduction electrons. However, it has scarcely been exploited for emergent function related to heat-electricity conversion. Here we report an unusually enhanced thermopower by application of magnetic field in MnGe hosting topological spin textures. By considering all conceivable origins through quantitative investigations of electronic structures and properties, a possible origin of large magneto-thermopower is assigned to the strong energy dependence of charge-transport lifetime caused by unconventional carrier scattering via the dynamics of emergent magnetic field. Furthermore, high-magnetic-field measurements corroborate the presence of residual magnetic fluctuations even in the nominally ferromagnetic region, leading to a subsisting behavior of field-enhanced thermopower. The present finding may pave a way for thermoelectric function of topological magnets.

## Abstract (web summary)

Topological spin textures produce versatile electronic functionalities, but are scarcely exploited for achieving heat to electricity conversion. Here, Fujishiro et al. attribute an enhanced magneto-thermopower in MnGe with topological spin hedgehogs, to electron scattering via the dynamics of an emergent magnetic field.

## Introduction

High-performance thermoelectric materials provide a viable solution towards environmental issues since they realize efficient electricity generation from waste heat without greenhouse gas emissions^1^. In long history of the research, extensive efforts have been made to enhance Seebeck coefficient (*S*) with minimal increase in electrical resistivity (*ρ*) to improve thermoelectric figure of merit *ZT* = *S*^2^*T*/*ρκ*, where *T* and *κ* represent temperature and thermal conductivity, respectively.

Seebeck coefficient, i.e., electromotive force per unit temperature gradient, can be interpreted as averaged entropy flow per charge carrier on the basis of the Onsager relations^2^. In the framework of the band structure picture, semi-classical treatment using the Boltzmann transport equation provides a guiding principle for obtaining the efficient entropy flow, which is described by Mott formula^1^; by neglecting the *T*-dependence of chemical potential *μ*, i.e., setting *μ* = *ε*_F_, it reads

1 $$S = - \frac{{\pi ^2k_{\mathrm{B}}^2T}}{{3e}}\left[ {\left. {\frac{{\partial \ln D\left( \varepsilon \right)}}{{\partial \varepsilon }}} \right|_{\varepsilon = \varepsilon _{\mathrm{F}}} + \left. {\frac{{\partial \ln \tau \left( \varepsilon \right)}}{{\partial \varepsilon }}} \right|_{\varepsilon = \varepsilon _{\mathrm{F}}}} \right],$$

where *k*_B_ and *e* are Boltzmann constant and elementary charge, respectively. The first and second terms in the brackets, respectively, represent energy derivatives of density of states *D*(*ε*) and relaxation time *τ*(*ε*) at Fermi energy *ε*_F_. It commonly occurs in metals that entropy flows of electrons with their potential energy above and below *ε*_F_ cancel out with each other, resulting in small *S* on the order of a few μV K^−1^. This is because the Fermi distribution function permits only electrons within their energy range approximately between *ε*_F_ ± *k*_B_*T* to be involved in heat transport phenomena, and electrons above and below *ε*_F_ carry heat (product of entropy and temperature) with opposite signs. The Mott formula suggests that such cancellation can be avoided in the presence of difference in number, velocity, and scattering rate between electrons above and below *ε*_F_, the first two of which are measured by the derivative $\left. {\frac{{\partial \ln D\left( \varepsilon \right)}}{{\partial \varepsilon }}} \right|_{\varepsilon = \varepsilon _{\mathrm{F}}}$ and the last of which by $\left. {\frac{{\partial \ln \tau \left( \varepsilon \right)}}{{\partial \varepsilon }}} \right|_{\varepsilon = \varepsilon _{\mathrm{F}}}$. Indeed, asymmetric band structures around *ε*_F_ generate large *S* (e.g., pseudogap structures in Heusler compounds^3^), whereas Kondo scattering creates strong energy dependence in *τ* and the consequent exotic Seebeck effect in rare-earth compounds^4^.

Partly because materials with good thermoelectric properties (e.g., Bi_2_Te_3_) have recently been identified as topological insulators^1,5^, the nontrivial electronic topology is expected to offer a unconventional mechanism to enhance thermoelectric effects. However, their good performance does not seem to be related to their topology, but can be understood in the context of the semi-classical model^6,7^. It also remains largely unexplored to utilize the emergent magnetic field acting on conduction electron, which is generated by topological spin structures in real or momentum space^8,9^, for improving thermoelectric efficiency. For one thing, that would be because existence of magnetic field would rather suppress *S* basically by quenching the internal degrees of freedom of carriers, such as spin and orbital entropy, spin-dependent scatterings, and so on, as exemplified by the observed reduction of *S* in Na_*x*_CoO_2_, where spin and orbital degeneracy is lifted by magnetic fields^10,11^. Suppression of *S* by magnetic field is also observed as a hallmark of chiral anomaly in Weyl semimetals such as GdPtBi where charge pumping causes dramatic decrease in *S*^12^.

**Fig. 1:**
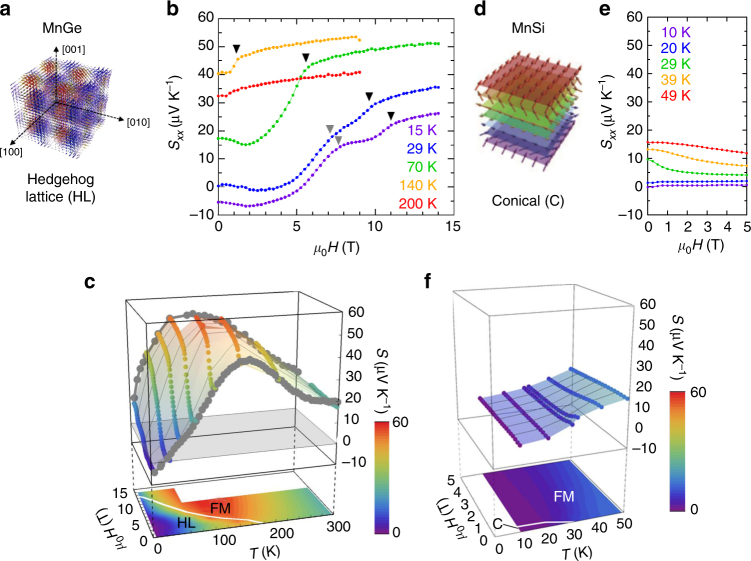
Schematics of spin textures and magneto-thermopower in MnGe and MnSi. **a** Spin texture of MnGe which can be regarded as a periodic array of hedgehogs and anti-hedgehogs with a short magnetic period of 3 nm. They serve as quantized source (monopole) and sink (anti-monopole) of emergent magnetic field, respectively. **b** Field-induced large magneto-thermopower observed in MnGe. Topological phase transition from hedgehog lattice to ferromagnetic state manifests itself as a kink in the *S*–*H* curve (shown as black triangles), suggestive of its link to the observed thermopower. There are also observed anomalies in the *S*–*H* curves at low temperatures, indicated by gray triangles. **c** Magneto-thermopower profiles for MnGe in the temperature–magnetic field space. The contour mappings are displayed in the bottom plane with the white line representing the phase boundary between ferromagnetic (FM) state and hedgehog lattice (HL). **d** Conical spin texture (C) with a magnetic period of 18 nm in MnSi. **e** Magneto-thermopower in MnSi as the contrasting example to MnGe, which shows a monotonic decrease generically found in magnetic materials. **f** Magneto-thermopower profiles for MnSi in the temperature–magnetic field space

B20-type compounds of the present focus, e.g., MnSi, (Fe,Co)Si, FeGe, and MnGe, form the family of chiral-lattice magnets hosting the magnetic skyrmion^13,14^. Noncentrosymmetric crystal structure allows Dzyaloshinskii–Moriya interaction beside ferromagnetic and other competing magnetic interactions, which in consequence gives rise to the universal phase diagram composed of helical/conical (Fig. 1d), ferromagnetic, and skyrmion phases^13^. MnGe shows a unique topological spin texture and occupies a unique position among those B20-type compounds. The skyrmion crystal is in general described by the multiple-**q** state, where **q** is the modulation vector of the helix state. The ordinary skyrmion crystal, e.g., in MnSi, is composed of the three **q**-vectors, which lie in the same plane and make the angle of 120° with each other, forming the two-dimensional triangular lattice. By contrast, the spin texture in MnGe is approximately described by the orthogonal three **q**-vectors along <100> directions and hence forms a three-dimensional lattice of spin hedgehogs and anti-hedgehogs (Fig. 1a). The spin hedgehog and anti-hedgehog therein serve as source (monopole) and sink (anti-monopole) of the emergent magnetic field acting on conduction electrons^8,9,15^. The large emergent magnetic field (~40 T) due to its short magnetic period (~3 nm) is already detected by transport measurements as the large topological Hall effect^16^ and the topological Nernst effect^17^. Another key difference from other B20-type chiral magnets is the extraordinarily large temperature (*T*)-magnetic-field (*H*) range (*T < T*_N_ ~ 170 K and *μ*_0_ |*H*|* < μ*_0_*H*_c_ ~ 12 T), including at 0 T, where the topological spin texture is realized as the thermodynamically stable magnetic state^16^. These features make MnGe a special material for seeking unprecedented physics originating in the topological spin texture.

Here we report the observation of magnetic-field-induced large thermopower in a chiral-lattice magnet MnGe with hedgehog-type topological spin textures^15,16,18^. The unprecedented *H*-dependence of *S* is highlighted by a comparative study of isostructural and isovalent magnet MnSi with helical/conical or skyrmion spin textures^19,20^, which exhibits usual monotonous decrease in *S* with application of magnetic fields^21^. Energy dependence of *D*(*ε*), which is revealed by photoemission spectroscopy (PES) and band calculations, shows some structures contributing to *S*, however, which alone is not enough to explain the observed magnitude. Along with other striking contrast between magneto-resistivity (MR) and specific heat of MnGe and MnSi, a unique scattering mechanism originating from strong fluctuations of emergent fields in MnGe may cause the strong dependence of transport lifetime, leading to the enhanced *S* even at low temperatures. This proposed scenario is corroborated by high-magnetic-field measurements, where we demonstrate the close correlation between MR and *S* in terms of magnetic fluctuations.

## Results

### Field-induced large thermopower in MnGe

We have observed unprecedented magneto-thermopower in the polycrystalline MnGe, which exhibits strong enhancement with increasing *H* below *T*_N_ (Fig. 1b). Its increment ratio becomes prominent at low temperatures: *S* shows a typical value for ordinary metals at zero field (e.g., −5.5 μV K^−1^ at 15 K), and it develops an order of magnitude larger at 14 T (e.g., 26 μV K^−1^ at 15 K). Phase transition from hedgehog lattice (HL, Fig. 1a) to ferromagnetic (FM) state at the critical magnetic field *H*_c_ is recognized as a kink in the *S*–*H* curve (indicated by the black triangle in Fig. 1b), followed by a saturating behavior. This clearly indicates the strong correlation between thermoelectric property and spin texture in this compound. By contrast, the thermopower in MnSi decreases with *H* (Fig. 1e), which is a behavior generically expected for magnetic materials; this highlights the unconventional magneto-thermopower in MnGe.

We also noticed some anomalous behaviors of *S* at low temperatures in MnGe; it keeps on increasing even above 14 T, although the spin texture should have almost turned into the FM state. The suppression of *S* is, however, eventually realized in the low-*T* and high-*H* region, along with the reduction of positive MR, which we attribute to the suppression of magnetic fluctuations caused by annihilation of HL. This will be discussed in a later section. Another point is that non-monotonous structure appears around 6–8 T (shown as gray triangles in Fig. 1b), however, these anomalies gradually disappear with the elevation of *T*. We speculate that this anomalous structure may be related to the *H*-dependence of emergent magnetic field in MnGe, whose magnitude becomes the maximum around the corresponding magnetic field^16^.

Comparison between *T*–*H* variations of *S* in MnGe and MnSi gives a clear summary of the features listed above, as shown in Fig. 1c, f. In MnGe, we can confirm the increasing behavior of thermopower towards the phase boundary between HL and FM (a white line in Fig. 1c) at every *T* and the saturating behavior in the FM state. The profile of *S* forms a broad peak structure around the phase boundary; this suggests a widespread effect of the large fluctuations around the HL–FM phase boundary, where the topological transition occurs as accompanied by the annihilation of hedgehog and anti-hedgehog magnetic textures^15^. A former study showed that hedgehog (anti-hedgehog) is viewed as emergent magnetic monopole (anti-monopole) and that the HL–FM topological transition corresponds to the pair annihilation of monopole and anti-monopole^15^. In contrast, MnSi shows nearly featureless profile of *S* (Fig. 1f) and no discernible structure in its narrow skyrmion phase region (see Supplementary Figure 1, Supplementary Note 1, and ref. ^21^).

### Electronic structure and thermopower

**Fig. 2:**
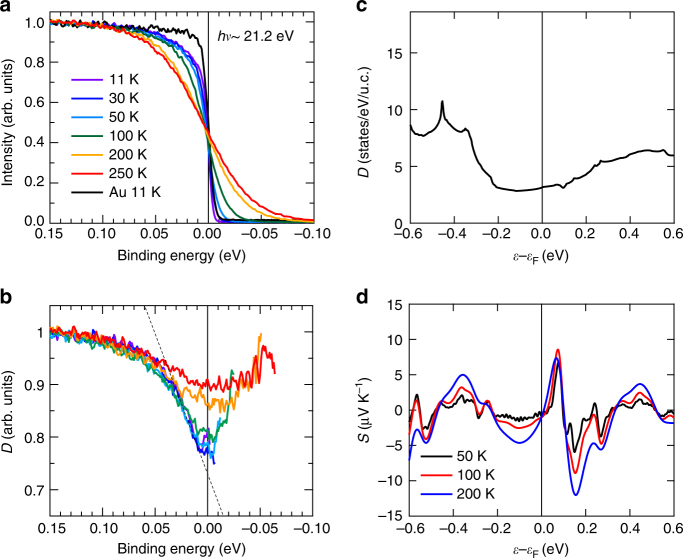
Electronic structure for MnGe. **a** Temperature dependence of photoemission spectra for MnGe with the reference spectrum of Au. **b** Effective density of states obtained by dividing the photoemission spectra by resolution-convoluted Fermi distribution function. Here the narrow pseudogap (~40 meV) is discernible especially below the transition temperature. The dashed line is a fitted linear function used for the estimation of thermopower. **c** Calculated density of states (*D*) of MnGe per unit cell (u.c.) assuming ferromagnetic state. **d** Seebeck coefficient calculated on the basis of the corresponding band structure in ferromagnetic state, which is far short of the experimental value. Vertical thin lines indicate the position of Fermi energy

**Fig. 3:**
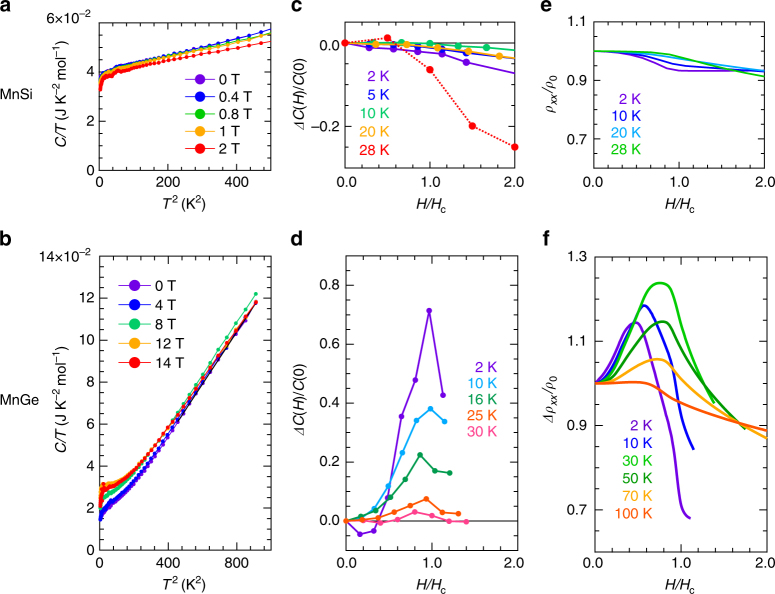
Specific heat and magneto-resistivity in MnGe and MnSi. Specific heat and magneto-resistivity for MnSi (**a**, **c**, **e**) and MnGe (**b**, **d**, **f**). **a**, **b** *C*/*T* plotted against *T*^2^ under various magnetic fields. *C* is the specific heat. **c**, **d** Change in the specific heat *ΔC*(*H*) as a function of external magnetic field normalized by the critical field *H*_c_ at respective temperatures. MnGe shows a sharp enhancement at the topological phase transition (*H*/*H*_c _= 1.0). **e**, **f** Magneto-resistivity normalized by the value at zero field at respective temperature. Large positive anomaly observed in MnGe (**f**) is attributed to the fluctuations of emergent magnetic field (monopole fluctuations)

To discuss what physical parameters mainly contribute to the large *S* in terms of the Mott formula, electronic structures and other magneto-transport properties have been investigated for MnGe. First, we examine the band structure of MnGe by performing photoemission spectroscopy (PES) and band calculation. Figure 2a shows *T*-dependence of photoemission spectra of MnGe near the Fermi level *ε*_F_. When a metallic system has a large Fermi surface with tiny energy dependence of *D*(*ε*), the spectrum obeys Fermi distribution function with respect to *ε*_F_. In the case of MnGe, deviation from the typical Fermi distribution function profile (represented by the spectrum in Au, indicated by a black line in Fig. 2a) becomes discernible with decreasing temperature. To further evaluate this *T*-dependence, we divided the spectra by resolution-convoluted Fermi distribution function to obtain the effective *D*(*ε*) (Fig. 2b). Here the formation of narrow pseudogap (~40 meV) is clearly observed especially below the transition temperature of MnGe (*T*_N_ ~ 170 K), suggesting its relationship with magnetic ordering in this system. We estimated the upper limit of its contribution to *S* by assigning the steepest downward slope of *D*(*ε*) curve at 11 K to the first term of Mott formula $- \frac{{\pi ^2k_{\mathrm{B}}^2T}}{{3e}}\frac{{\partial \ln D\left( \varepsilon \right)}}{{\partial \varepsilon }}$ (see the dashed line in Fig. 2b for the corresponding slope). It turns out that the narrow pseudogap generates thermopower of *S* *≈* 0.13 *T* (μV K^−1^). Under the assumption that application of *H* effectively causes a shift in *ε*_F_ while keeping the pseudogap structure robust, our estimation of the upper limit of *S* should be also valid for the FM state; the estimated value of *S* is far short of the experimental one. We also calculated the band structure in the FM state using the density functional theory (DFT) to theoretically derive *S* (see Methods). As shown in Fig. 2c, *D*(*ε*) does not present any distinct structures like the pseudogap detected by PES. Therefore the corresponding *S* calculated with the approximation of constant relaxation time represents only a small value of –0.9 μV K^−1^ at 50 K (Fig. 3d). Thus, electronic structure of MnGe alone cannot be the dominant source for the unconventional thermoelectric response as observed.

### Specific heat and magneto-resistivity

The field-induced behavior of *S* is suggestive of enhancement of entropy, which can be best illustrated by the field-dependent specific heat (*C*). Temperature dependence of *C* under various *H* in MnSi and MnGe are shown as the curves of *C*/*T* vs. *T*^2^ in Fig. 3a and b, respectively. We also convert these data to the change in ratio of specific heat [*ΔC*(*H*)/*C*(0) = (*C*(*H*)—*C*(0))/*C*(0)] as functions of normalized magnetic field (*H*/*H*_c_) at various temperatures (Fig. 3c for MnSi and Fig. 3d for MnGe). There is again stark contrast between these two systems. As to MnSi, we observe a monotonous decrease of specific heat with *H* at every temperature. Here we note that the significant decrease in *ΔC*(*H*)/*C*(0) is due to release of latent heat associated with the first-order transition from the conical to collinear (ferromagnetic) spin structure (see Fig. 3c, 28 K and ref. ^22^). In contrast, specific heat of MnGe shows a clear increase around *H*_c_, where the spin hedgehogs and anti-hedgehogs undergo the pair annihilation and the topological transition into the FM state occurs^15^. There obviously exist strong fluctuations unique to the topological phase transition in MnGe. Now we can estimate the maximum of thermoelectric contribution from the entropy enhancement in thermal equilibrium. If we can take full advantage of the increase in specific heat *ΔC*(*H*), *S* can change by *ΔS*(*H*) = *ΔC*(*H*)/*ne*, whatever the mechanism is (e.g., phonon or magnon drag^23^). With the largest *ΔC* (0.181 J K^−1^ mol^−1^ at 25 K) and carrier density *n* (~1.3 × 10^23^ cm^−3^)^16^, we obtain *ΔS*(*H*) ≤ 0.52 μV K^−1^, which is again quantitatively insufficient to be a dominant origin of the large magneto-thermopower in MnGe.

As all the above possible origins have failed to explain the large *H*-induced *S* in MnGe, some scattering processes are likely to make a major contribution, producing a large value of the second term in Mott formula $- \frac{{\pi ^2k_{\mathrm{B}}^2T}}{{3e}}\frac{{\partial \ln \tau \left( \varepsilon \right)}}{{\partial \varepsilon }}$. In other words, the carriers should acquire the large entropy by getting scattered. With increasing *H*, such *H*-enhanced scattering of carriers manifests itself in a large positive MR around the *H*-induced HL–FM topological transition, as observed in a previous study^15^ and reproduced for the present sample in Fig. 3f. Note that enhanced spin fluctuations accompanied by those of the emergent magnetic field around the topological phase transition, are evidenced also by the result of specific heat (Fig. 3d). The characteristic *H*-dependence of MR in MnGe (Fig. 3f) shows a broad peak structure around *H*_c_ at every temperature below *T*_N_. This positive MR is also unconventional since external magnetic fields basically suppress spin-dependent scattering, which leads to the monotonously decreasing negative MR as observed in MnSi (Fig. 3e) and other related B20-type compounds^24^. The anticipated strong energy dependence of *τ* may also be rooted in such a *H*-dependent enhancement of the fluctuating emergent magnetic field.

### Magneto-resistivity and thermopower at the low-temperature and high-field regime

**Fig. 4:**
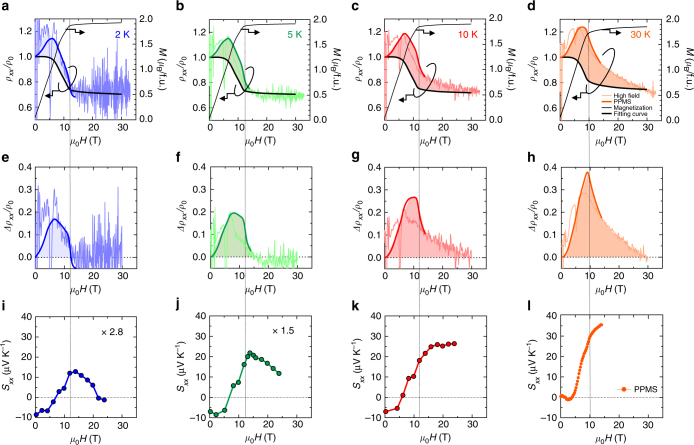
Magneto-resistivity and thermopower in MnGe at high-magnetic fields. **a**–**d** Longitudinal magneto-resistivity (MR) measured by pulsed magnet at low temperatures (*T* = 2, 5, 10, 30 K). Thick-line curves are the results on the same sample by steady-field measurements (PPMS) up to 14 T. Bold black curves are the estimated conventional MR associated with the variations of magnetization shown in the black thin lines. Large noises, in particular for the low-field and low-temperature region, in the pulse-field results are due to the low resistivity of the sample, <10 μΩ cm. **e**–**h** The estimated positive MR contributions due to the emergent-field fluctuations, which correspond to the color-shaded regions in **a**–**d**. **i**–**l** Magnetic-field dependence of thermopower at low temperatures measured with a 25 T superconducting magnet (*T* = 2, 5, 10 K) and PPMS (*T* = 30 K)

The positive MR due to the fluctuations of emergent field only gradually falls down and still remains well above *H*_c_ as shown in Fig. 3f. This implies that there may exist robust or pinned excitations of spin states with non-coplanar spin arrangements like hedgehogs even in the FM phase, causing large magnetic fluctuations as a source of the non-diminishing behavior of *S*. To corroborate this interpretation, we performed high-magnetic-field measurements on MR (up to 33 T) and *S* (up to 24 T) at low temperatures (below 10 K), where such magnetic fluctuations is anticipated to be sufficiently suppressed. Figure 4a–d shows the results for MR and *M*. As the resistivity is generally expressed as $\left[ {W \cdot \tau } \right]$^−1^ with *W* being Drude weight, the MR can be decomposed to the field-induced respective changes of *W* and* τ*. The MR due to the field-change of *W* is well known for the double-exchange system (e.g., colossal magnetoresistance manganites^25^) and can be well scaled with the magnetization *M*, as confirmed also for the present case of MnGe^15^. Here, with use of the corresponding magnetization data, we estimate the conventional negative MR due to the field-increase of *W*, as shown with black lines in Fig. 4a–d. Then, we can deduce the effect of magnetic fluctuations on MR, i.e., the field-induced change of *τ*^−1^, as the deviation from the conventional negative MR^15^. The estimated deviations, which correspond to the color-shaded regions of Fig. 4a–d, are displayed in Fig. 4e–h. They clearly show the residual magnetic fluctuations to scatter the conduction electrons in the FM state, which appear to be steeply enhanced with increasing temperature across *T* = 10 K. As for *S* in the high-field regime, we found a strong correlation with the observed MR. A decreasing behavior of *S* with the field is clearly identified across the HL to FM transition (shown in dashed lines) for *T* = 2 K and 5 K (Fig. 4i, j) along with the strong suppression of magnetic fluctuations as evidenced by the MR measurement (Fig. 4e, f). When the temperature is elevated, by contrast, the non-decreasing behavior of *S* (Fig. 4j–l) takes over, due to the finite magnetic fluctuations surviving in the FM phase (Fig. 4f–h). Even a larger magnetic field (more than 24 T) seems to be required to fully suppress the enlarged magnetic fluctuations above 5 K. Here we note that the enhancement of *S* does not measure the variation of scattering rate *τ*^−1^ itself but its energy dependence $S \propto \partial {\mathrm{ln}}\tau /\partial \varepsilon$ as described by Mott’s formula. Hence, the enhancement of *S* can happen in principle as long as there exist any finite magnetic fluctuations (Fig. 4f–h) affecting *τ*, although the quantitative connection between *τ* and $\partial \tau /\partial \varepsilon$ is difficult to verify at the moment.

### Thermoelectric power factor

We lastly note that *S* and thermoelectric conversion efficiency [power factor *S*^2^/*ρ* (μW K^−2 ^cm^−1^)] largely vary in different samples. The maximum power factor obtained in our study reaches as large as *S*^2^/*ρ* = 65 μW K^−2 ^cm^−1^ at *H* = 14 T, *T* = 19 K, although it shows the large sample dependence and is apparently related to the value of residual resistivity or to the background (*H* = 0) transport lifetime (Supplementary Figures 2 and 3).

## Discussion

We have unraveled an unusual magneto-Seebeck effect in MnGe, which shows a large enhancement by applying external magnetic field. Through examining its origin from every possible aspect by photoemission spectroscopy, band calculation, specific heat, and magneto-transport measurements, we propose that the anomalous enhancement is rooted in strong energy dependence of transport lifetime *τ*, which may arise from the *H*-dependent dynamics of emergent magnetic field. In addition, high-field measurements on MR and *S* verifies the presence of surviving magnetic fluctuations to scatter the conduction electrons even in the FM phase. The most important integrant for the observed thermoelectric phenomena in MnGe should be the dense lattice of magnetic singularities like spin hedgehogs and anti-hedgehogs, where their large emergent fields and fluctuations critically affect the motion of electrons. The paradigm presented in this paper, that is the efficient heat-electricity conversion of topological origin, may lead to new guiding principles of achieving high thermoelectric performance in topological magnets.

## Methods

### Sample preparation

Polycrystalline samples of MnGe were prepared by high-pressure synthesis technique. Mn and Ge were first mixed with an atomic ratio of 1:1 and then melted in an arc furnace under an argon atmosphere. Afterwards, it was heated at 1073 K for 1 h under 4 GPa in a cubic-anvil-type high-pressure apparatus. Powder X-ray analyses confirmed B20-type crystal structure (*P*2_1_3) with no detectable impurity content (Supplementary Figure 4). A single crystal of MnSi was grown by the Czochralski method in tetra-arc furnace under an argon atmosphere. Powder X-ray diffraction pattern of the pulverized single crystal indicated that the sample was of single phase.

### Thermoelectric measurement

The samples of MnGe and MnSi were cut into rectangular shape with the size of about 4 × 2 × 0.5 mm^3^. Magnetic field was applied along the longest side of sample ([111] direction for the MnSi single crystal), which is parallel to the thermal current generated by 1 kΩ chip resistor. The temperature gradient was read by two Cernox thermometers attached to the sample with varnish. Voltage was measured through Manganin wires attached to the sample by solder pastes. Temperature and magnetic field were controlled by Physical Property Measurement System (PPMS), Quantum Design. The high-field measurements of thermopower were performed utilizing 25 T Cryogen-free Superconducting Magnet (CSM) installed at High Field Laboratory for Superconducting Materials of Institute for Materials Research (IMR), Tohoku University, Japan^26^.

### Transport and specific heat measurements

Magneto-resistivity and specific heat capacity were measured by using AC-transport option (AC excitation current of 23 Hz and 20 mA) and heat capacity option, respectively, with Physical Property Measurement System (PPMS). Magnetic field was applied parallel to electrical current for magneto-resistivity measurement. High-field measurements of magnetization and longitudinal magneto-resistivity were performed utilizing nondestructive pulsed magnets installed at International MegaGauss Science Laboratory of Institute for Solid State Physics (ISSP), University of Tokyo, Japan. Magnetization was measured by the conventional induction method, using coaxial pickup coils. Resistivity was measured by the conventional four probe method with voltage pre-amplifiers using a numerical lock-in technique with an excitation current of 25 kHz and 20 mA.

### Photoemission spectroscopy

Photoemission spectroscopy on MnGe was performed with a VG-Scienta R4000WAL electron analyzer and a helium discharge lamp with the photon energy of 21.2 eV at the University of Tokyo. The energy resolution was set to 8 meV. The Fermi energy was determined from the photoemission spectrum of a gold film evaporated on the substrate, within an accuracy of better than ± 0.3 meV. The MnGe sample was fractured at 11 K in an ultrahigh vacuum better than 1 × 10^−10^ Torr. We confirmed the reproducibility of the temperature-dependent photoemission spectrum by the temperature-cycled measurements.

### Calculations of band structure and Seebeck coefficient

Electronic structure calculations for ferromagnetic MnGe were performed using the density functional theory with a generalized gradient approximation^27^ as implemented in the quantum-ESPRESSO code^28^. Ultrasoft pseudopotentials^29^ and the plane-wave basis set with cutoff energies of 50 Ry for wave functions and 400 Ry for charge densities were used. Seebeck coefficients were obtained within the semi-classical Boltzmann theory using wannier90 code^30,31^ (See Supplementary Figure 5 for the calculated band structure).

### Data availability

The data sets generated during and/or analyzed during the current study are available from the corresponding author on reasonable request.

## Electronic supplementary material

Supplementary Information
