# Supplementary material for: Large magneto-thermopower in MnGe with topological spin texture
Source: Nat Commun. 2018 Jan 29;9:408. doi: 10.1038/s41467-018-02857-1 (PMC5789084; doi:10.1038/s41467-018-02857-1)
Supplement: Supplementary file 1 — Supplementary Information [file 41467_2018_2857_MOESM1_ESM.pdf]

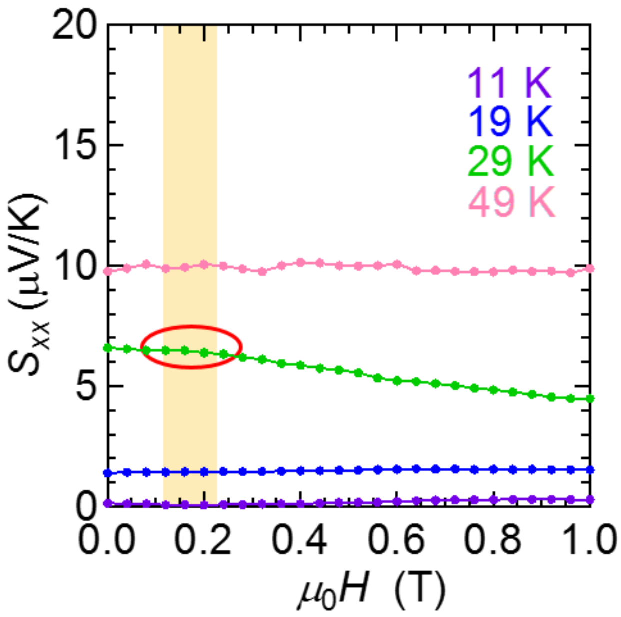

Supplementary Figure 1 | Magnetic field dependence of thermopower in MnSi with skyrmion lattice (SkL). Magnetic field dependence of thermopower (*S*) at various temperatures. No anomaly in *S* is discernible in the yellow-shaded region, where SkL is realized at 29 K. Magnetic field is applied perpendicular to the temperature gradient in this case.

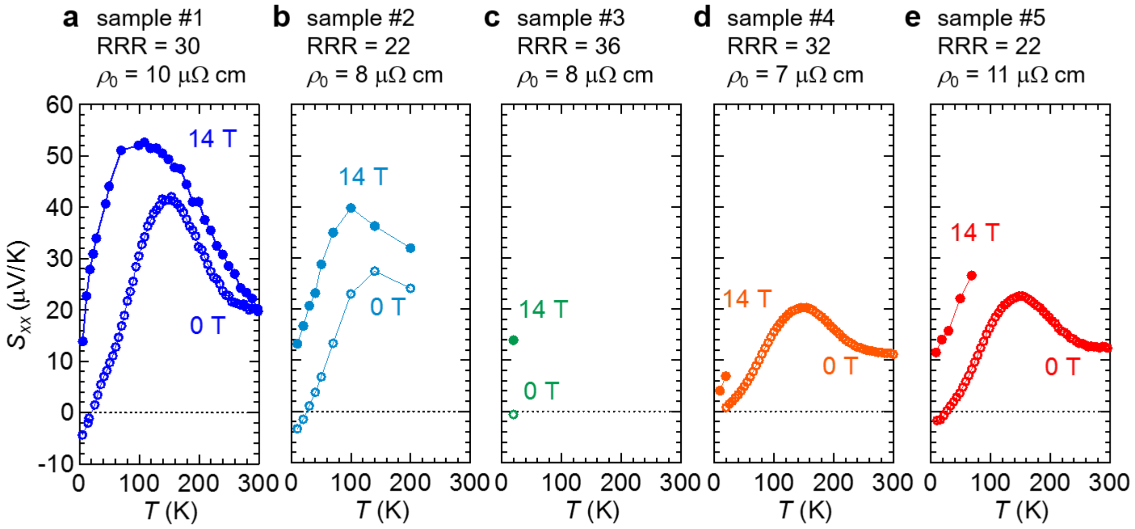

Supplementary Figure 2| Temperature dependence of thermopower for several samples with different residual resistivity ratio [RRR = ρ(300 K)/ρ(2 K)]. a, Thermopower of the sample which is presented in the main text. **b-d**, Thermopower of other samples with various RRR for comparison.

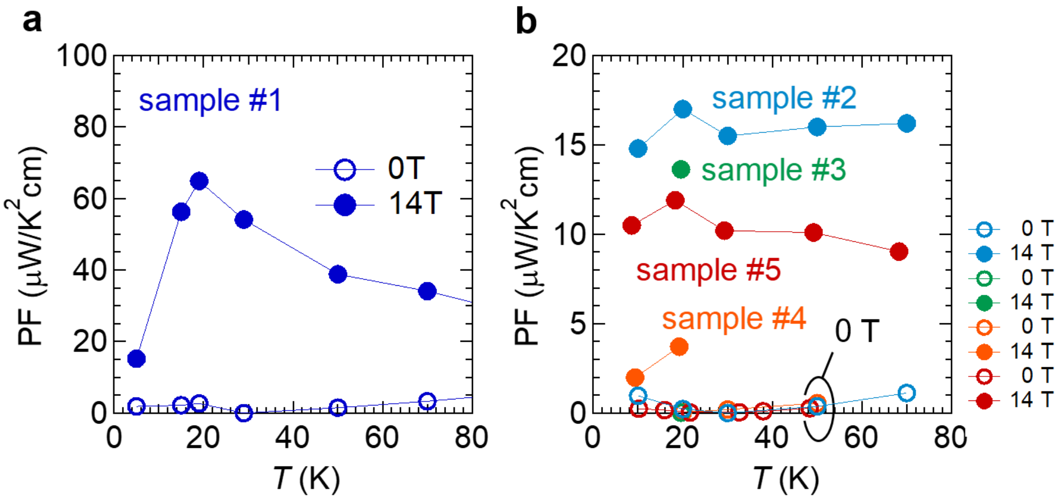

Supplementary Figure 3 | Temperature dependence of power factor (PF) for MnGe and its sample dependence. a, Temperature dependence of PF (= S^2/ρ) at 0 T and 14 T for sample #1 which exhibits the largest thermopower at low temperatures. **b**, Temperature dependence of PF at 14 T for various samples with different sample quality.

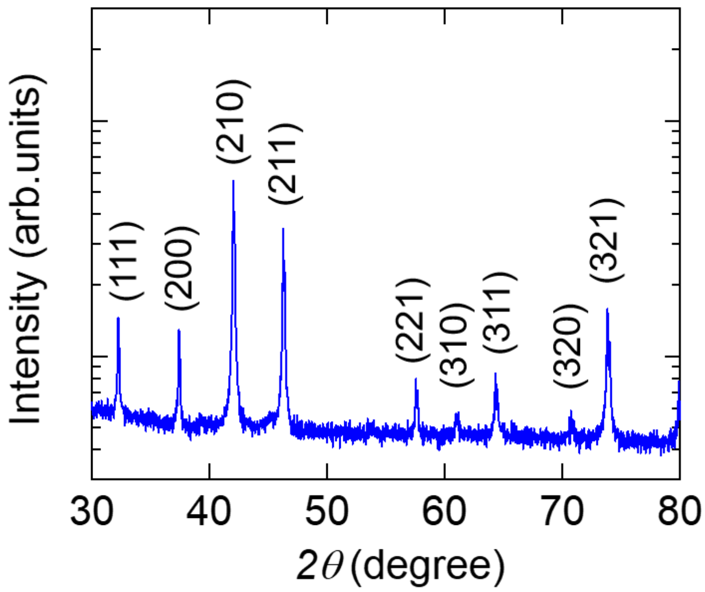

Supplementary Figure 4 | X-ray diffraction pattern of MnGe. Polycrystalline MnGe crystallizes into B20-type crystal structure (space group $P2_13$). No impurity peak is discernible, which indicates the high sample quality.

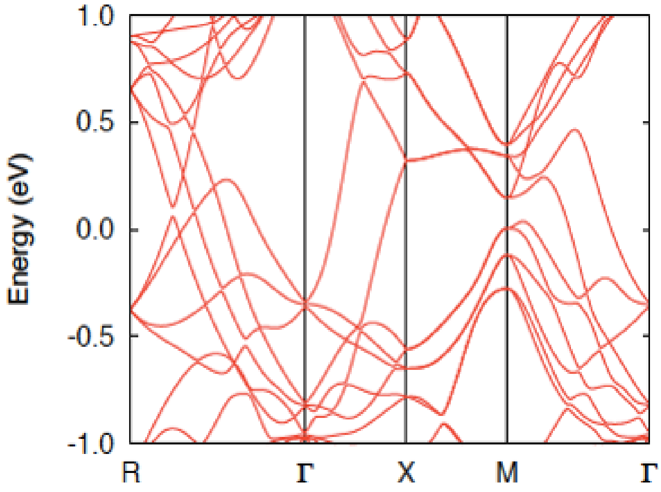

Supplementary Figure 5 | Band structure in the ferromagnetic state for MnGe.

Supplementary Note 1 │Comment on the absence of enhancement of thermopower in the skyrmion lattice phase.

We comment on the possible reasons why the enhancement of S due to carrier scattering related to SkL is indiscernible in MnSi. The possible reason is that the effect of scattering due to SkL on transport properties may be too small to be observed because the effective emergent magnetic field B_{eff} (~theoretically 11 T) in MnSi is one order of magnitude smaller than that of MnGe (~theoretically 400 T) due to the large difference in their magnetic period λ_{mag}, *i.e.*, the density of topological spin textures ($B_{\mathrm{eff}} \propto 1/\lambda_{\mathrm{mag}}^2$). Instead, a minute *suppression* of S (less than 1.5%) in the SkL of MnSi is reported by S. Arsenijević *et al.*, which is attributed to the reduced entropy caused by the formation of the ordered SkL[1]. Hence even if there were any positive contribution of S from the carrier scattering upon the melting of SkL, which is presumably tiny, it would be compensated by the negative contribution of magnetic ordering (e.g. conical-ferromagnetic ordering) effect, resulting in the suppression of S.

Supplementary References

1. Arsenijević, S., Petrovic, C., Forro, L. & Akrap, A. Manifestation of the spin textures in thermopower of MnSi. *Europhys. Lett.* **103**, 57015 (2013).
